# New insights into the mechanisms of the immune microenvironment and immunotherapy in osteosarcoma

**DOI:** 10.3389/fimmu.2024.1539696

**Published:** 2025-01-17

**Authors:** Cong Luo, Xingxing Min, Danying Zhang

**Affiliations:** ^1^ Department of Orthopedic Trauma, Zhuji People’s Hospital of Zhejiang Province, Zhuji, Zhejiang, China; ^2^ Department of Emergency and Critical Care, Shanghai Changzheng Hospital, Shanghai, China

**Keywords:** osteosarcoma, immune microenvironment, immunotherapy, immune evasion, combination therapy

## Abstract

Osteosarcoma, a malignant bone tumor primarily affecting adolescents, is highly invasive with a poor prognosis. While surgery and chemotherapy have improved survival for localized cases, pulmonary metastasis significantly reduces survival to approximately 20%, highlighting the need for novel treatments. Immunotherapy, which leverages the immune system to target osteosarcoma cells, shows promise. This review summarizes the biological characteristics of osteosarcoma, mechanisms of pulmonary metastasis, and the tumor immune microenvironment (TME). It involves recent immunotherapy advances, including monoclonal antibodies, tumor vaccines, immune cell therapies, checkpoint inhibitors, and oncolytic viruses, and discusses combining these with standard treatments.

## 1 Introduction

Osteosarcoma is a heterogeneous malignant tumor affecting bone and soft tissues, primarily in children and adolescents, with high invasiveness and a strong tendency for pulmonary metastasis (1, 2). Surgery alone results in a five-year survival rate of about 20%, but chemotherapy increases this rate to 70% (3). However, the prognosis remains poor once metastasis occurs, particularly to the lungs (4). The biological characteristics of osteosarcoma largely arise from genetic mutations in mesenchymal stem cells (MSCs), such as in P53 and RB1 genes, which increase the risk of MSCs transforming into malignant cells (5, 6). Osteosarcoma cells express Runx2 and Sox9 genes, showing features of osteoblastic and chondrogenic differentiation (7). Ewing sarcoma’s origin remains controversial, with potential sources including neural crest stem cells, embryonic progenitors, or MSCs (8). Osteosarcoma exhibits significant genetic heterogeneity, with around 7-14% of patients harboring actionable mutations, particularly in the IGF signaling pathway (9). Genome-wide studies have identified genes and pathways involved in osteosarcoma progression and metastasis, including WNT/β-catenin, Notch, and CD99, emphasizing the importance of precision medicine in diagnosis and treatment (10).

Immunotherapy has emerged as a promising treatment for cancers (11–13), utilizing immune-mediated cytotoxic effects against tumors (14). The tumor immune microenvironment (TME) includes immune cells, cytokines like IL-6 and TNF-α, and regulatory factors, all of which contribute to tumor progression and metastasis (15). Pulmonary metastasis in osteosarcoma involves the activation of WNT/β-catenin and Notch pathways, high expression of ezrin, and cytokines like TGF-β and IL-6/IL-8, which facilitate cell invasion and migration (16). Immune checkpoint inhibitors and cell therapies have shown potential in osteosarcoma treatment, though challenges remain, including treatment variability, adverse reactions, and high costs (17, 18). This review highlights the biological characteristics, molecular mechanisms of pulmonary metastasis, and progress in immunotherapy, exploring the clinical potential and challenges to inform more effective treatment strategies for osteosarcoma.

## 2 Classification and biological characteristics of osteosarcoma

Osteosarcoma is a heterogeneous malignancy primarily affecting bone and soft tissues, commonly seen in children and adolescents. Its pathogenesis involves genetic mutations in MSCs, notably in P53 and RB1 genes, which promote malignant transformation (2). Osteosarcoma and chondrosarcoma cells express Runx2 and Sox9 genes, indicating osteogenic and chondrogenic differentiation, respectively (7). Ewing sarcoma’s cellular origin remains debated, with possible derivations from neural crest stem cells, embryonic bone and cartilage progenitors, or MSCs. Fusion proteins in Ewing sarcoma complicate its classification (19). While impaired MSC differentiation is believed to contribute to osteosarcoma and chondrosarcoma, the exact mechanisms are still unclear.

At the molecular level, osteosarcoma shows significant genetic heterogeneity, with about 21% of patients harboring actionable mutations, especially in the IGF signaling pathway. Genome-wide association studies (GWAS) have identified susceptibility loci, including the GRM4 gene (6p21.3) and a gene desert region at 2p25.2 (20). High-grade osteosarcoma samples also show mutations in TP53, RB1, and 82 other genes. The TARGET-OS database has identified 12 survival-related genes, with eight downregulated (e.g., ERCC4, CLUAP1) and four upregulated (e.g., MUC1, JAG2) (21, 22). Recent studies highlight the role of various signaling pathways and genetic alterations in osteosarcoma progression. Weighted gene co-expression network analysis has linked osteosarcoma metastasis to pathways like microtubule formation, Cytochrome P450 drug metabolism, IL-17 signaling, DNA replication, cell adhesion, and heparin binding (23). Whole-transcriptome analysis reveals changes in extracellular matrix degradation and collagen biosynthesis (24). Additionally, CD99 suppresses osteosarcoma malignancy (25). These findings underscore the importance of genomic and transcriptomic analyses for uncovering osteosarcoma’s biological mechanisms and identifying new therapeutic targets for precision medicine.

## 3 Tumor immune microenvironment of osteosarcoma

TME is a complex system composed of immune cells, cytokines, and regulatory factors surrounding tumor cells. It plays a crucial role in osteosarcoma initiation, progression, and metastasis (26). This section explores the immune cells, regulatory factors, immune suppression and activation mechanisms, and tumor cell strategies to evade immune surveillance within the osteosarcoma TME.

### 3.1 Immune cells and immune regulatory factors

The osteosarcoma TME includes a variety of immune and non-immune cells, with stromal cells playing a key role in expressing EMT genes, which are linked to immune responses (27, 28). Alaa et al. found that stromal cells secrete cytokines promoting EMT, increasing tumor invasiveness and metastatic potential (29). Osteosarcoma stem cells, due to their chemoresistance, plasticity, and immune modulation abilities, contribute to metastasis and immune evasion (30). Several immune-related genes and cytokines are crucial in the TME (31–34). Liang et al. developed a three-gene risk model (TYROBP, TLR4, and ITGAM), regulating macrophage activation and predicting patient outcomes (35). Lipid metabolism genes were linked with the TME, suggesting their potential as prognostic biomarkers (36–38). Cytokines like IL-6 are pivotal in immune evasion and chemoresistance. Huang et al. identified IL-6’s role in promoting cell proliferation and anti-apoptotic mechanisms via the STAT3 signaling pathway (39). Additionally, mutations in P53 and RB1 within the TME can influence the behavior of immune cells (40). P53 mutations can lead to an immunosuppressive microenvironment by upregulating PD-L1 expression, thereby facilitating immune escape (41). RB1 mutations may enhance the recruitment of myeloid-derived suppressor cells (MDSCs), further contributing to immune evasion and promoting a tumor-friendly environment (42–44). These findings highlight the importance of immune regulatory factors in the osteosarcoma TME and their potential as therapeutic targets.

### 3.2 Interactions between osteosarcoma cells and immune cells

Single-cell RNA sequencing (scRNA-seq) and multi-omics has revealed the complexity of the TME (45–50). Huang et al. identified the diverse spatial distribution and functional states of immune cells in the osteosarcoma TME (51). Chen et al. found that lipid metabolism gene expression is closely linked to the TME, serving as reliable prognostic biomarkers (52). These studies highlight the importance of immune cell distribution and gene expression in developing targeted therapies and improving patient outcomes. Interactions between osteosarcoma cells and immune cells are pivotal in tumor immune evasion and progression. While normal lymphocytes can exert cytotoxic effects on osteosarcoma cells *in vitro*, osteosarcoma cells can disrupt dendritic cell (DC) function, impairing immune responses (53, 54). Grzegorz et al. showed that osteosarcoma cells secrete IL-10, inhibiting DC maturation and antigen presentation (55). Additionally, osteosarcoma cells interact with host cells and immune responses at multiple levels (56). These interactions provide insights into osteosarcoma pathogenesis and suggest potential targets for immune-based therapies. Audrey et al. found that osteosarcoma cells secrete TGF-β, which suppresses T cell activity and aids immune evasion (57).

### 3.3 Immune suppression and activation in osteosarcoma

Osteosarcoma is often a “cold tumor” with limited immune cell infiltration, leading to immune suppression through upregulated factors like PD-L1 (58). Despite this, some studies suggest that immune activation is possible using immune checkpoint inhibitors. Park et al. demonstrated that PD-1 inhibitors enhanced T cell cytotoxicity against osteosarcoma cells (17). Additionally, osteosarcoma cells suppress immune responses by modulating CXCL12 (59). Neoadjuvant chemotherapy can transform osteosarcoma into an immunologically “hot” tumor, activating the local immune environment. Myrofora et al. found that chemotherapy increased T cell infiltration in osteosarcoma, suggesting it promotes immune activation, creating potential for immunotherapeutic strategies (60).

### 3.4 Immune evasion mechanisms

Osteosarcoma cells evade immune responses through extracellular matrix alterations, immune suppressive pathways, and high PD-L1 expression, which inhibit T cell activity (17). Osteosarcoma cells upregulate PD-L1 as a strategic mechanism to evade immune surveillance, thereby facilitating tumor progression and resistance to therapeutic interventions (61). Additionally, TGF-β promotes regulatory T cell (Treg) expansion, further suppressing immunity (62). Targeting immune evasion mechanisms offers promising strategies. Dong et al. showed that inhibiting TGF-β reduced osteosarcoma cell invasiveness (63). Combining PD-L1 and TGF-β inhibitors enhanced immune cell cytotoxicity against osteosarcoma, underscoring the potential of combination therapies to overcome immune escape (64).

## 4 Immunotherapy strategies for osteosarcoma

**Figure 1:**
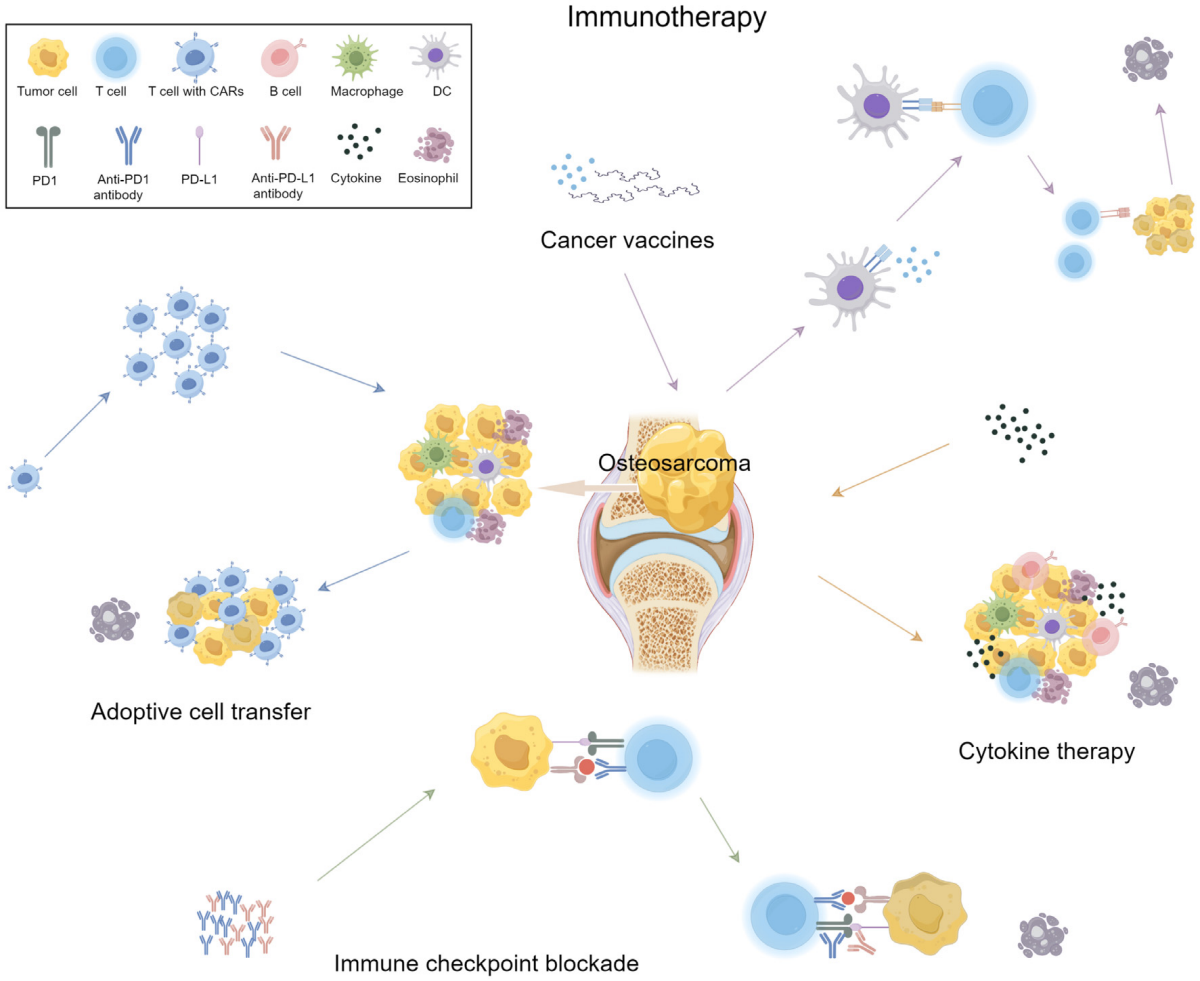
Clinical immunotherapy in Osteosarcoma.

Immunotherapy offers a more targeted approach to cancer treatment compared to traditional chemotherapy, which generally attacks rapidly dividing cells (65). The immune system, through processes like immune surveillance and cell infiltration, plays a crucial role in fighting cancer (66). However, tumor cells can evade immune responses through mechanisms such as immune editing, which includes three phases: elimination, equilibrium, and escape (67). In the elimination phase, immune cells target and destroy cancer cells. In the equilibrium phase, some tumor cells survive and adapt, entering a dormant state. Eventually, these cells may escape immune detection and proliferate (68, 69). Mechanisms of immune escape include loss of tumor antigens, downregulation of HLA expression, recruitment of immune-suppressive cells like Tregs and M2 macrophages, and the upregulation of immune checkpoint receptors such as CTLA-4 and PD-1 (70–72). Immunotherapy seeks to counteract these escape mechanisms by boosting the immune system’s ability to recognize and destroy tumor cells (**Figure 1**).

### 4.1 Antibody-based therapies targeting cell surface proteins

Osteosarcoma cells express specific surface antigens that are potential targets for immunotherapy (17). Monoclonal antibodies can bind to these antigens, activating NK cells and macrophages to release cytotoxic substances, leading to tumor destruction via antibody-dependent cellular cytotoxicity (ADCC) (73, 74). For instance, Persaud et al. (75) demonstrated the efficacy of antibody therapy in neuroblastoma, suggesting similar potential in osteosarcoma. Bispecific T-cell engagers (BiTEs), which bind both T cell CD3 receptors and tumor antigens, enhance T cell activation and cancer cell lysis (76). Holzmayer et al. showed that bispecific antibodies boosted T cell-mediated osteosarcoma cell killing (77). Additionally, antibody-drug conjugates (ADCs) link antibodies to cytotoxic agents like vedotin, targeting cancer cells with higher specificity and efficacy (78). Antibody-based therapies can cause infusion reactions (fever, chills, allergies), cardiotoxicity, neurotoxicity, infections, requiring careful monitoring and supportive care (79).

### 4.2 Tumor vaccines

Tumor vaccines function by exposing or administering tumor antigens to induce tumor-specific immune responses, thereby enabling the immune system to recognize and attack tumor cells. These vaccines come in various forms, including whole tumor cells, lysates, proteins, DNA, RNA, and peptides (80). DCs are pivotal antigen-presenting cells capable of activating T cells and stimulating the proliferation of cytotoxic T lymphocytes (CTLs) (81). For instance, Lu et al. (3) developed DC vaccines by combining tumor cell lysates with immunostimulatory adjuvants, significantly enhancing immune-mediated cytotoxicity against osteosarcoma in patients. Moreover, the development of personalized tumor vaccines, such as those based on patient-specific tumor mutations, is emerging as a critical component of precision medicine (82). These vaccines offer new avenues for osteosarcoma immunotherapy by providing tailored immune responses against unique tumor antigens. The integration of personalized vaccines into clinical practice holds promise for improving treatment outcomes and patient survival rates. Adverse reactions to tumor vaccines are generally mild, including injection site inflammation and systemic symptoms, though rare severe immune-mediated events may occur (83). Ongoing research aims to mitigate these effects through enhanced vaccine design and delivery.

### 4.3 Immune cell therapy

Immune cell therapy is a promising approach for metastatic and recurrent osteosarcoma, particularly when combined with neoadjuvant chemotherapy. Neoadjuvant chemotherapy activates the local immune milieu, transforming osteosarcoma into an immunologically “hot” tumor, thereby enhancing the efficacy of subsequent immunotherapies (59). Wang et al. observed increased T cell infiltration in the tumor microenvironment post-chemotherapy, improving immune responses (84). Mifamurtide has shown clinical efficacy as adjuvant therapy for non-metastatic osteosarcoma, indicating that immune checkpoint inhibitors may significantly improve prognosis (85). Additionally, immune-related gene expression diagnostics could support personalized treatments (86). Challenges include patient selection and managing immune-related adverse effects (87). Phase I/II trials are addressing these to enhance safety and efficacy while elucidating the tumor microenvironment’s role in osteosarcoma pathogenesis (88). Integration of immunotherapies, such as mifamurtide and checkpoint inhibitors, holds substantial potential for better outcomes (89). Adoptive cell therapy, including Chimeric antigen receptor T-cell (CAR-T) therapy targeting HER2 (90), NK cells and tumor-infiltrating lymphocytes (TILs) enhances anti-tumor immunity by overcoming immune escape (91, 92). These findings highlight the potential of adoptive cell therapy in osteosarcoma treatment, though clinical application requires further optimization. However, immune cell therapies may induce cytokine release syndrome, neurotoxicity, and autoimmunity (93), which can be mitigated through monitoring, safety switches, and supportive care.

### 4.4 Checkpoint inhibitors

Immune checkpoint inhibitors block inhibitory signals between tumor and immune cells, reactivating T cell-mediated anti-tumor responses. Osteosarcoma cells often upregulate immune checkpoint molecules like PD-L1, which suppress T cell activity and facilitate immune escape (15). Studies show that anti-PD-1 and anti-PD-L1 antibodies significantly improve survival in osteosarcoma mouse models and reduce pulmonary metastasis (94). Zheng et al. found that anti-PD-1 antibody treatment effectively controlled lung metastases in osteosarcoma models (95). Combining checkpoint inhibitors with chemotherapeutic agents (e.g., doxorubicin) is an effective strategy, as chemotherapy can reverse the tumor’s immunosuppressive state, enhancing inhibitor efficacy (96). Additionally, combining checkpoint inhibitors with radiotherapy shows potential, though more clinical evidence is needed. Overall, immune checkpoint inhibitors offer substantial promise for osteosarcoma treatment, particularly in prolonging survival and addressing pulmonary metastasis. However, optimizing and personalizing their use remains an important area for future research. Checkpoint inhibitors, while effective, induce immune-related adverse events across multiple organs (97), necessitating immunosuppression and careful monitoring.

### 4.5 Oncolytic virus therapy

Oncolytic virus therapy employs genetically engineered viruses that selectively replicate within and lyse malignant cells, representing an innovative immunotherapeutic approach. These viruses not only exert specific cytotoxic effects on tumor cells but also promote an inflammatory response within the tumor microenvironment, enhancing antigen presentation and the maturation of antigen-presenting cells, thereby boosting the immune system’s ability to recognize and attack tumors (98, 99). Recently, several genetically modified oncolytic viruses have shown promise in preclinical and clinical trials for osteosarcoma. Herpes simplex virus (HSV), a complex double-stranded DNA virus, has been genetically modified (e.g., G207 and NV1020) to enhance its selective oncolytic activity against tumor cells while minimizing damage to normal cells (100). Neeti et al. reported that G207 exhibited significant oncolytic activity in osteosarcoma cell lines and effectively inhibited tumor growth in animal models (101). Similarly, adenoviruses (e.g., VCN-01) and modified Delta-24-RGD oncolytic adenoviruses have demonstrated potent anti-tumor effects, capable of suppressing primary osteosarcoma growth and preventing metastasis (102, 103). Additionally, vaccinia virus (VV), known for its efficient replication and large genome capacity, has shown considerable potential in tumor therapy (104). Morales et al. found that genetically modified VV exhibited significant anti-tumor efficacy in osteosarcoma models, further validating its feasibility as an emerging immunotherapeutic agent (105). In summary, oncolytic virus therapy, as an innovative immunotherapeutic approach, has demonstrated favorable safety and efficacy profiles in osteosarcoma treatment, supporting its further clinical application and research. Adverse reactions to oncolytic virus therapy include flu-like symptoms, injection site reactions, rare systemic inflammation (e.g., myocarditis, cytokine release syndrome), and potential viral transmission, necessitating strict biosafety protocols (106).

### 4.6 Combination therapy strategies

Combination therapies are increasingly recognized as essential for osteosarcoma treatment, integrating traditional approaches such as chemotherapy, radiotherapy, and surgery with emerging immunotherapies (107). This integrative strategy enhances therapeutic efficacy, reduces recurrence, and improves survival. For instance, a recent phase II clinical trial combined PD-L1 inhibitors with doxorubicin-based chemotherapy, demonstrating a synergistic effect that improved response rates and overcame chemoresistance in osteosarcoma patients (108). Another study combined CTLA-4 inhibitors with targeted therapies against the IGF signaling pathway, resulting in enhanced tumor regression and reduced metastatic spread (109). These examples illustrate how combination therapies can effectively address the complex resistance mechanisms inherent in osteosarcoma. Additionally, combining PD-L1 antibodies with chemotherapy agents can reverse chemotherapy-induced immunosuppression, boosting the immune system’s ability to target tumor cells (110). Additionally, trials involving the combination of immune checkpoint inhibitors with PARP inhibitors have shown promising results in preclinical models, suggesting potential for overcoming DNA repair-related resistance in osteosarcoma. Clinical trials by Zhang et al. demonstrated improved response rates with this combination (111). Demethylation pretreatment combined with immunotherapy also shows promise, with Wang et al. finding that demethylating agents enhance immune recognition and treatment efficacy (112). Furthermore, integrating oncolytic virus therapy with checkpoint blockade has enhanced antigen presentation and T cell infiltration, overcoming the immunosuppressive tumor microenvironment (113). Other novel therapies, including inhalation therapy, targeted radiotherapy, and antibody therapy, improved survival when combining targeted radiotherapy with antibody therapy (114).

Multimodal approaches incorporating surgery and radiotherapy have shown significant benefits, such as reducing tumor recurrence and enhancing survival rates in jaw osteosarcoma (114). Furthermore, the presence of P53 and RB1 mutations may influence the efficacy of combination therapies. For instance, tumors harboring P53 mutations may exhibit resistance to certain chemotherapeutic agents, necessitating the inclusion of targeted immunotherapies to overcome this resistance (115). Similarly, RB1 mutations may enhance the metastatic potential of osteosarcoma cells, making it imperative to integrate therapies that specifically address metastatic pathways alongside conventional treatments (116). Overall, combination therapy offers more comprehensive, personalized treatment options, improving therapeutic outcomes by integrating traditional and novel approaches and significantly reducing recurrence and mortality rates. However, combination therapies may exacerbate adverse reactions, including myelosuppression and organ toxicities (117, 118). This underscores the imperative for meticulous monitoring and the development of individualized treatment protocols to mitigate such risks effectively.

## 5 Conclusion

Immunotherapy strategies for osteosarcoma encompass a range of approaches, including antibody-based therapies, tumor vaccines, immune cell therapies, adoptive cell therapies, checkpoint inhibitors, and oncolytic virus therapies. By thoroughly understanding the distribution and roles of immune cells within the tumor immune microenvironment, the mechanisms of immune regulation, and the strategies employed by tumor cells to evade immune responses, researchers can develop more precise and effective immunotherapeutic protocols. Although immunotherapy has shown substantial promise in the treatment of osteosarcoma, several challenges remain, such as the realization of personalized treatment, management of immune-related adverse effects, and control of treatment costs. Future research should focus on optimizing immunotherapy strategies, exploring the best combinations for multimodal therapy, and validating their safety and efficacy through clinical trials. These efforts are essential to advancing immunotherapy for osteosarcoma, ultimately improving clinical outcomes and the quality of life for patients.
